# A simple framework to analyze water constraints on seasonal transpiration in rubber tree (*Hevea brasiliensis*) plantations

**DOI:** 10.3389/fpls.2014.00753

**Published:** 2015-01-06

**Authors:** Jessada Sopharat, Frederic Gay, Philippe Thaler, Sayan Sdoodee, Supat Isarangkool Na Ayutthaya, Charlchai Tanavud, Claude Hammecker, Frederic C. Do

**Affiliations:** ^1^Department of Plant Science, Faculty of Natural Resources, Prince of Songkla UniversitySongkhla, Thailand; ^2^UMR Eco&Sols, French Agricultural Research Centre for International Development (CIRAD)Montpellier, France; ^3^Hevea Research Platform in Partnership, DORAS Centre, Kasetsart UniversityBangkok, Thailand; ^4^Department of Plant Science and Agricultural Resources, Faculty of Agriculture, Khon Kaen UniversityKhon Kaen, Thailand; ^5^Department of Earth Science, Faculty of Natural Resources, Prince of Songkla UniversitySongkhla, Thailand; ^6^UMR Eco&Sols, Institut de Recherche pour le DéveloppementMontpellier, France

**Keywords:** potential transpiration model, drought, high evaporative demand, canopy phenology, relative extractable soil water

## Abstract

Climate change and fast extension in climatically suboptimal areas threaten the sustainability of rubber tree cultivation. A simple framework based on reduction factors of potential transpiration was tested to evaluate the water constraints on seasonal transpiration in tropical sub-humid climates, according pedoclimatic conditions. We selected a representative, mature stand in a drought-prone area. Tree transpiration, evaporative demand and soil water availability were measured every day over 15 months. The results showed that basic relationships with evaporative demand, leaf area index and soil water availability were globally supported. However, the implementation of a regulation of transpiration at high evaporative demand whatever soil water availability was necessary to avoid large overestimates of transpiration. The details of regulation were confirmed by the analysis of canopy conductance response to vapor pressure deficit. The final objective of providing hierarchy between the main regulation factors of seasonal and annual transpiration was achieved. In the tested environmental conditions, the impact of atmospheric drought appeared larger importance than soil drought contrary to expectations. Our results support the interest in simple models to provide a first diagnosis of water constraints on transpiration with limited data, and to help decision making toward more sustainable rubber plantations.

## Introduction

The rubber tree (*Hevea brasiliensis*) is a major tree crop in Southeast Asia. Globally, it covers 9.82 million hectares mainly exploited by smallholders (UNCTAD, 2013). The tree is a native from the rainforests of the equatorial region of the Amazon basin (Priyadarshan et al., 2005). Traditionally, it has been cultivated in the equatorial belt and humid zones with a tropical and monsoonal climate (Raj et al., 2005). Currently, Thailand is the world's top producer of natural rubber. The plantations are mainly located where conditions are optimal in southern Thailand. However, in the last 30 years, the rubber plantations have largely expanded into climatically sub-optimal areas in north and northeast Thailand. The dynamic of land use is similar to that in other rubber-producing countries, with expansions also recorded in northeast India, the highlands and coastal areas of Vietnam, southern China, and the southern plateau of Brazil (Priyadarshan et al., 2005). In such areas, rubber can be constrained by drought, low temperature and high altitude or conversely by periodic heavy rainfall. Moreover, with climate change, higher frequencies of extreme events (flooding, drought) in the rainy season and an increase in temperature and evaporative demand in the dry season are expected in both traditional and new areas (Masaki et al., 2011). In addition, despite the large extension of land covered by rubber plantation, little is known of its environmental impacts and particularly about carbon and water balances (Guardiola-Claramonte et al., 2010; Kumagai et al., 2013). To address the sustainability of rubber plantations and to choose appropriate plant material and management practices, it is necessary to forecast rubber tree behavior on a large scale and over long periods of time. Hence the availability of simple models with limited data to analyze water constraints on tree transpiration and consequences on growth, production and soil water balance is a key issue (Guardiola-Claramonte et al., 2010; Boithias et al., 2012; Carr, 2012).

Our final objective is to evaluate, on an annual basis, the relative contributions of soil water shortage and atmospheric drought to the regulation of maximal transpiration in rubber tree stands under various pedoclimatic conditions. The use of a robust and simple model based on reduction factors of potential transpiration simulation appeared as a reasonable first approach to schematically separate the main controls. Granier et al. (1999) proposed such a model of daily water balance called BILJOU to evaluate water constraints in forest stands. It has been successfully used in temperate and tropical humid forests (Granier et al., 1999; Wagner et al., 2011). Transpiration models based on canopy conductance regulation have been also assessed and used (Jarvis, 1976; Stewart, 1988; Granier et al., 2000, 2007). However, they require an hourly step, more parameters and input data. As a first approach we chose to use the framework of BILJOU99 (Granier et al., 1999). The model assumes, under non-limiting soil water, a linear response of maximal transpiration (*T*_max_) vs. potential evapotranspiration (*PET*) for a leaf area index (*LAI*) inferior to 6, the slope being the ratio “*rm*” depending on the *LAI*. The model assumes that under a soil water shortage, *rm* decreases linearly below a threshold of relative extractable water (*REW*) of 0.4. Like many models, it does not consider direct constraint due to atmospheric drought (Boote et al., 2013). However, Isarangkool Na Ayutthaya et al. (2011) have shown that transpiration in mature rubber trees was strongly regulated above a threshold of evaporative demand, whatever the soil water availability. Such a regulation was related to an isohydric behavior expressed by stability of minimum leaf water potential and maximum whole-tree hydraulic conductance. The idea was to add a reduction factor above a critical climatic demand.

To test our modeling approach based on BILJOU99 framework, we selected a representative mature stand where trees faced the full range of soil and atmospheric drought conditions over a complete annual cycle. Tree transpiration, the evaporative demand and the soil water availability were measured every day over 15 months. The first objective was to test simplified controls of transpiration through evaporative demand, leaf area index and relative extractable water. We hypothesized that the basic relationships hold, except when the evaporative demand becomes too high. The second objective was to test an evolution of the model including sensitivity to atmospheric drought. We assumed that it would provide a reasonable indication of the trend in seasonal transpiration. The third objective was to assess by modeling the hierarchy between soil water and atmospheric constraints on transpiration on an annual basis. We assumed a predominant control by soil drought according to common thinking.

## Materials and method

### Study site

The plantation was located at Baan Sila (N15° 16′ 23″ E103° 04′ 51.3″), Khu-Muang, Buri Ram province in northeast Thailand. The experiments were conducted in a monoclonal plot (clone RRIM 600), planted at 2.5 × 7.0 m spacing (571 trees ha^−1^). The trees were 11 years old and had been tapped for 4 years for latex harvesting. The soil is deep with a loamy sand texture. The mean contents of clay, loam and organic matter varied from 9.9, 24.2, to 0.78% in the surface layer (0–0.2 m) to 20.2, 23.6, and 0.34% at a depth of 1.5 m, respectively. In this non-traditional rubber tree plantation area, the environmental conditions are water limiting for *H. brasiliensis*. The dry season lasts 6 months, from November to April, and average annual rainfall is 1176 mm. Canopy yellowing and defoliation occurred between December and March. In a sample of 237 trees, canopy fullness was assessed every 2 weeks for each tree according to seven categories of the percentage of green leaves (100, 90, 75, 50, 25, 10, and 0%). When the defoliation was almost complete, the maximum leaf area index (*LAI*_*max*_) was estimated from leaves collected in nine 1 m^2^ litter traps. A schematic change in the *LAI* over the year was deduced from observations of canopy fullness and litter fall measurements.

### Climatic measurements

The local microclimate was monitored automatically in an open field at a distance of 50 m from any trees. An automatic weather station (Minimet automatic weather station, Skye Instruments Ltd, Llandrindod Wells, UK) recorded half-hourly values of air temperature, relative humidity, incoming short wave radiation, wind speed and rainfall. The reference evapotranspiration was calculated according to the FAO 56 formula in Allen et al. (1998).

### Soil water content measurements

**Figure 1:**
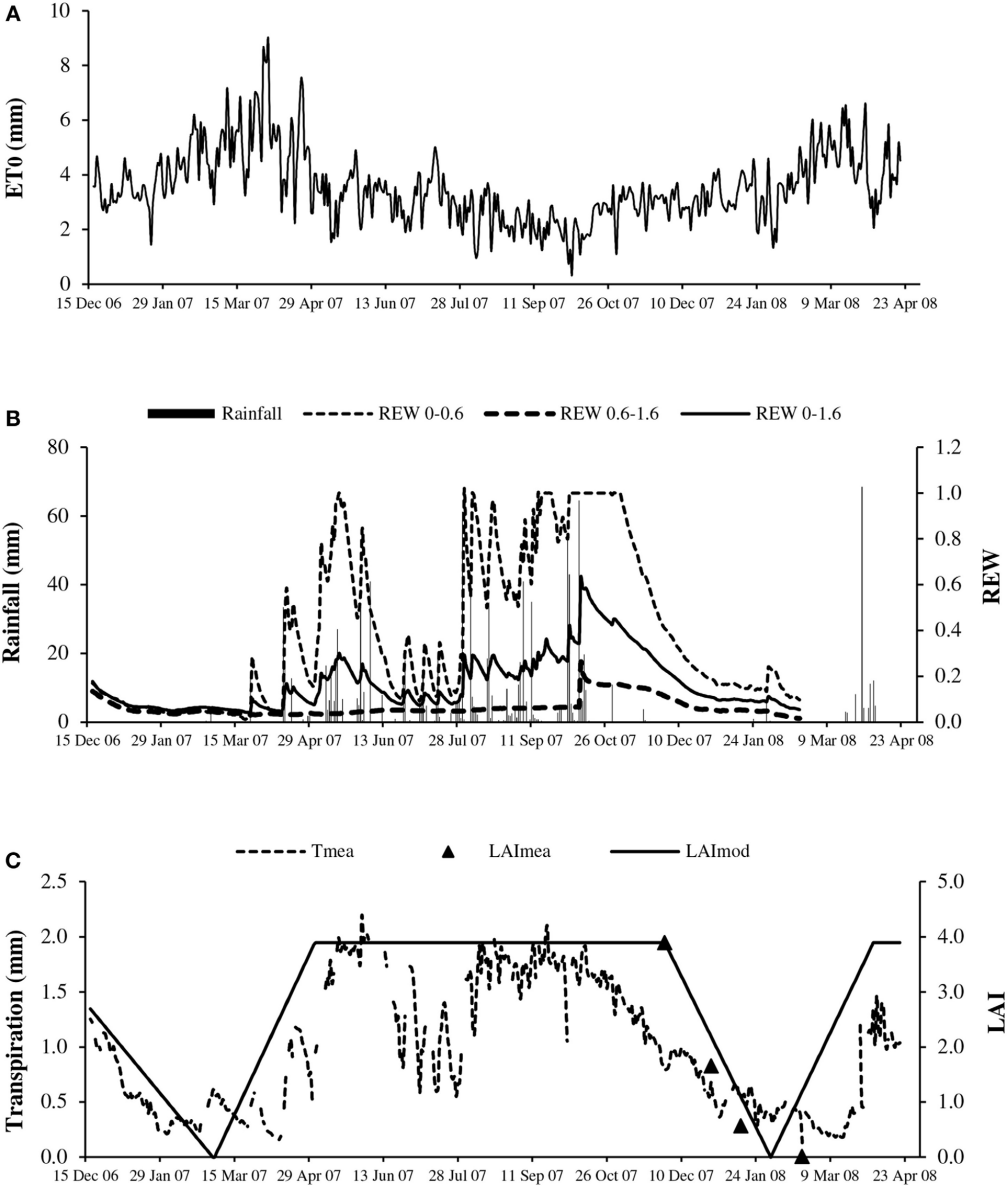
**Seasonal course of: (A) reference evapotranspiration (*ET0*); (B) rainfall (solid bar) and relative extractable water of bulk soil (*REW*) for layers 0–0.6 m (*REW* 0–0.6), 0.6–1.6 m (*REW* 0.6–1.6) and 0–1.6 m (*REW* 0–1.6); and (C) measured transpiration (*T*_mea_, dotted line) and leaf area index estimated from litterfall (*LAI*_mea_, triangles) and schematic shape (*LAI*_mod_, solid line)**.

Volumetric soil water content (θ) was measured with a neutron probe (3322, Troxler, Research Triangle Park, NC, USA) calibrated for the experimental soil with separate calibrations for the upper (0–0.2 m) and lower (below 0.2 m) layers. Twelve 2 m-long tubes were set up in pairs; in each pair, one tube was located in the planting line between two trees, and the other in the middle of the inter-row. Measurements were made every 0.2 m, from a depth of 0.1 to 1.5 m every 2 weeks. Based on observed fluctuations in soil water, the soil profile was separated into two layers: topsoil (0–0.6 m) and subsoil (0.6–1.6 m). The average field capacity and permanent wilting points were measured as 0.21 and 0.07 m^3^ m^−3^ for the topsoil, and 0.25 and 0.10 m^3^ m^−3^ for the subsoil, respectively (Isarangkool Na Ayutthaya et al., 2010). Additionally, θ was measured continuously with a capacitance probe (EnvironSCAN System, Sentek Sensor Technologies, Adelaide, SA, Australia). The vertical probe included nine sensors located every 0.2 m at the same level as the neutron probe measurements. For each sensor, θ was estimated from cross-calibration with the neutron probe measurements over the whole seasonal range. Relative extractable water was calculated for each layer according to Granier et al. (1999):

(1) $$REW_{i}=(W-W_{m})/(W_{f}-W_{m})$$

where *REW_i_* is the relative extractable water in each soil layer *i*, *W_m_* is the minimum soil water content, *W_f_* is the soil water content at field capacity and *W* is the actual soil water content. To calculate the total *REW* for the sensitive root zone, *REW_i_* was weighted by the percentage of fine root length within each layer. As the soil profiles showed low soil water availability and little change in the subsoil (Figure 1B), for modeling purpose the total *REW* was calculated for the top soil (0–0.6 m). In this site, the top soil contained 83% of the fine root length accumulated down to 1.6 m (Gonkhamdee et al., 2009). The fraction of fine root length used for weighting the *REW* in the top soil was 0.63, 0.32, and 0.05 for the layers 0–0.2, 0.2–0.4, and 0.4–0.6 m, respectively.

### Transpiration measurement

The xylem sap flow density was measured using the transient thermal dissipation method (TTD, Isarangkool Na Ayutthaya et al., 2010). The TTD method is based on using the same Granier probe design and heating power but uses a cyclic schedule of heating and cooling to assess a transient thermal index over a 10-min rise in temperature. The hourly sap flux density (*J*_*s*_; kg m^−2^ h^−1^) was calculated according to the non-species-specific calibration assessed by Isarangkool Na Ayutthaya et al. (2010):

(2) $$J_{s}=12.95\times K_{\text{a}}\times 10^{2}$$

where *K*_a_ is the transient thermal index. A temperature signal (Δ*T*_a_) was defined as

(3) $$\Delta T_{\text{a}}=\Delta T_{\text{on}}-\Delta T_{\text{off}}$$

where Δ*T*_on_ is the temperature difference reached at the end of the 10-min heating period and Δ*T*_off_ is the temperature difference before heating. To measure *J*_s_ every half hour with a heating period of 10 min, a cycle of 10 min heating and 20 min cooling was applied and the temperature signals were recorded every 10 min. Δ*T*_off_ wasinterpolated at the time of Δ*T*_on_ from Δ*T*_off_ surrounding measurements. The transient thermal index was calculated as:

(4) $$K_{\text{a}}=(\Delta T_{\text{0a}}-\Delta T_{\text{ua}})/\Delta T_{\text{ua}}$$

where Δ*T*_0a_ is the maximum temperature difference obtained under zero flow conditions and Δ*T*_ua_ is the measured signal at a given *J*_s_. the zero flux signal was determined every night assuming that sap flow was negligible at the end of the night. This assumption was strongly supported by slight change of daily Δ*T*_0a_ over the study period and minimum nocturnal *VPD* always lower than 0.3 kPa (Donovan et al., 2001). The probes were inserted into the trunks at a height of 1.8 m above the soil. At this height, average sapwood area was estimated to be 1.97 × 10^−2^ m^2^. After removal of the bark, the 2-cm-long probes were inserted to a depth of 2.5 cm into the sapwood, in such a way that the whole probe was fully inside the conductive sapwood. Three probes were inserted into each trunk to account for circumferential variability. The trunk area containing the probes was protected from direct solar radiation and rainfall by a deflector. Probes were connected to a data logger (CR10X, Campbell Scientific, Leicester, UK). Hourly sap flow density (*J*_s_), measured in the outermost ring of the sapwood, was accumulated over 24 h to calculate daily *J*_s_ (*J*_out_day_ expressed in kg m^−2^ d^−1^). To account for radial variation in the sap flux density in the deep sapwood, a reduction coefficient of 0.874 was applied to the *J*_s_ measured in the outermost ring of conducting xylem (Isarangkool Na Ayutthaya et al., 2010). Finally, neglecting tree water storage, transpiration (*T*; mm d^−1^) was estimated according to the equation:

(5) $$T=0.874\times 10^{-2}\times J_{\text{out}\_\text{day}}\times (\text{sapwood area/tree spacing area})$$

### Canopy conductance calculation

The canopy conductance (*G_c_*; mm s^−1^) was calculated by inverting an approximate of the Penman-Monteith equation. The approximation assumes that tree stand transpiration is well coupled to the atmosphere, i.e., decoupling coefficient (Ω) close to 0 (Jarvis and McNaughton, 1986; Phillips and Oren, 1998):

(6) $$G_{c}=\frac{\gamma \;\cdot \;\lambda \;\cdot \;T}{C_{p}\;\cdot \;\rho \;\cdot \;VPD}$$

where γ is the psychrometric constant (Pa K^−1^), γ is the latent heat of water vaporization (J kg^−1^), *T* is transpiration, *C*_*p*_ is the specific heat of dry air at constant pressure (J kg^−1^ K^−1^), ρ is the atmospheric density (kg m^−3^) and *VPD* is the air vapor pressure deficit (kPa). *G_c_* was calculated at midday (*G*_c_md_) from the maximum vapor pressure deficit (*VPD*_max_) and the daily maximum transpiration estimated from the sap flow (*T*_max_; mm s^−1^).

### Modeling

#### Basic relationships

The main relationships of BILJOU99 framework have been described in the introduction and more details are provided in Granier et al. (1999). The model inputs are daily data of leaf area index, rainfall and Penman potential evapotranspiration (*PET*). Instead of *PET* we used the FAO reference evapotranspiration *ET0* (Allen et al., 1998) which is currently available in world weather networks. The model can simulate the soil water balance by daily tipping buckets if runoff is negligible, which was not the case here. The present study was focused on transpiration controls and for the sake of accuracy we used only measured soil water availability.

#### Details of calculation

Potential, climatic or maximal transpiration was first calculated from the following equation:

(7) $$T_{\text{max}}\;=\;rm*ET0$$

where *rm* depends on *LAI*_max_;

Second, *T*_max_ could be decreased according to the *LAI* pattern by the following reduction coefficient:

(8) $$rLAI\;=\;(LAI/LAI_{\text{max}})$$

The effect of soil water shortage was simulated by the calculation of a reduction coefficient (*rREWc*) as:

(9) $$\begin{array}{l} rREWc=rLAI\;if\;REW>REWc\\ rREWc=(rLAI/REWc)*REW\;if\;REW\leq REWc \end{array}$$

where *REWc* is the critical value of relative soil water content.

Hence the transpiration was calculated according to the following formula:

(10) $$T_{\text{mod}}\;=\;rREWc*T_{\text{max}}$$

The expected saturation of transpiration at high evaporative demand was introduced in the BILJOU99 framework by applying a reduction coefficient above a critical value of *ET0*:

(11) $$T_{\text{mod}\_\text{ET0c}}\;=\;min(rREWc;rET0c)*T_{\text{max}}$$

For simplicity of writing we could have included the negative effect of high *ET0* in equation [7]. But we have preferred to separate the effects because of functional reasons. Equation [7] represents a general climatic driving effect (positive) on transpiration whatever plant species. While *rET0c* in equation [11] expresses a negative effect which varies according species and which is attributed to plant hydraulic limitations (Oren and Pataki, 2001; Bush et al., 2008; Isarangkool Na Ayutthaya et al., 2011; Ocheltree et al., 2014).

#### Diagnosis of hierarchy between water constraints

The approach was to use the calibrated framework to simulate independently or in combination the factors that control the regulation of transpiration on an annual basis and to separate the rainy season and dry season. The regulations of transpiration was expressed as the ratio between the cumulated potential transpiration, driven by evaporative demand and stand characteristics under full canopy conditions (*LAI*_max_), and the cumulated actual transpiration possibly reduced by defoliation, soil water availability or sensitivity to air dryness. Such a calculation assumes that reduction factors act independently which is certainly not true in the details. The annual cycle of transpiration was considered from January 1 to December 31, and the rainy season from May 1 to October 31. The few gaps in the daily transpiration were interpolated from *ET0* using an average ratio *T/ET0* measured on surrounding data (at least 4 days).

### Data analysis

Statistics were performed using the XLSTAT software (Addinsoft, Paris, France). The agreement between measured and simulated data was quantified by using the coefficient of determination (*R^2^*), root mean square error (*RMSE*) and relative root mean square error (*RRMSE*). Absolute and relative root mean square errors were calculated according to the following formulas:

(12) $$\begin{array}{l} \;RMSE=\sqrt{\frac{\sum_{i\;=\;1}^{n}{\left(x_{mea,i}-x_{\text{mod},\text{i}}\right)}^{2}}{n}},\\ RRMSE\;=\;\sqrt{\frac{\sum_{i\;=\;1}^{n}{\left(x_{mea,i}-x_{\text{mod},\text{i}}/x_{mea,i}\right)}^{2}}{n}} \end{array}$$

where *x*_*mea*, *i*_ is the measured value, x_mod, i_ is the simulated value at place *i* and *n* is the number of values.

## Results

### Seasonal variations of environmental water constraints, *LAI*, and transpiration

The evaporative demand as expressed by *ET0* largely fluctuated between 1 and 9 mm d^−1^ with a cumulative value of 1247 mm year^−1^ (Figure 1A), 23% above the cumulative rainfall (965 mm) in 2007. *ET0* values were particularly high during the 6 months of the dry season, from 3 mm d^−1^ in November up to 9 mm d^−1^ in March, corresponding to a *VPD* value above 1.0 kPa. The evaporative demand remained relatively high in the first part of the rainy season (from May to July) and decreased markedly in August, September and October, with *ET0* and *VPD* values below 2.0 mm d^−1^ and 0.7 kPa, respectively.

The water availability of the bulk soil or *REW* logically followed the rain occurrence (Figure 1B), with values close to 1.0 at the start of the rainy season in the topsoil (0–0.6 m). The availability decreased sharply to 0.2 in July, in the middle of the rainy season. The *REW* again reached high values (above 0.5) from August to October. *REW* values above 1 at the end of October suggested temporary water logging in the topsoil which was confirmed by observations. The *REW* quickly decreased in the dry season, reaching 0.2 in January. In the deep soil (0.6–1.6 m), the *REW* value indicated low water availability with little change over the year (maximum 0.26).

As represented by the schematic change in the *LAI* (Figure 1C), leaf shedding occurred in January and February, immediately followed by leaf flushing, with the latter occurring when the evaporative demand was the highest. The maximum *LAI* deduced from litterfall measurements averaged 3.9 at the end of 2007 (*n* = 9, *SD* = 0.7). The period with full canopy included approximately the period of highest soil water availability and lowest evaporative demand.

The maximum transpirations estimated by sap flow measurements (*T*_mea_) were steady (approximately 2.0 mm d^−1^) throughout the period with full canopy (Figure 1C). The intermittent decreases evident in June and July related to the evolution of the *REW* in the topsoil (Figure 1B). The lowest transpiration corresponded to the time of leaf shed. However, it never reached zero values despite almost complete defoliation. However, the sap flow measurement using the TTD method has low accuracy at very low flow rates (Isarangkool Na Ayutthaya et al., 2010). The minimum recorded transpiration was 0.1 mm d^−1^ on April 11, 2007 and the maximum value was 2.2 mm d^−1^ on May 31, 2007.

### Transpiration vs. *ET0, REW*, and *LAI*

#### ET0

**Figure 2:**
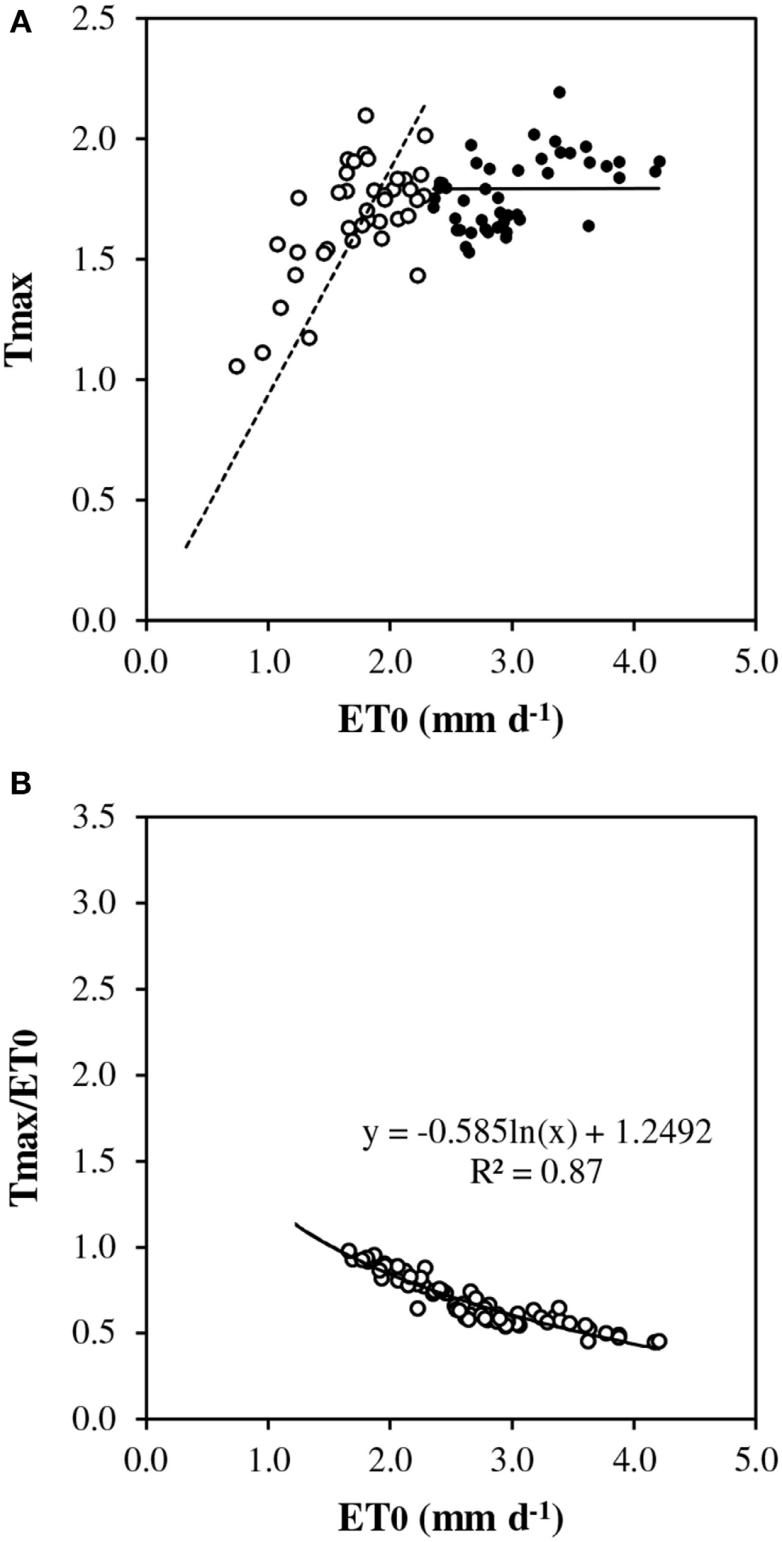
**Daily transpiration vs. reference evapotranspiration (*ET0*): (A) measured maximum transpiration (*T*_max_) in the absence of soil water stress (*REW* 0–0.6 ≥ 0.5), closed circles indicate *ET0* > 2.3 mm d^−1^, open circles indicate *ET0* < 2.3 mm d^−1^, dotted line represents the linear regression below 2.3 *ET0* and crossing origin; (B) relative transpiration (*T*_max_/*ET0*)**.

**Figure 3:**
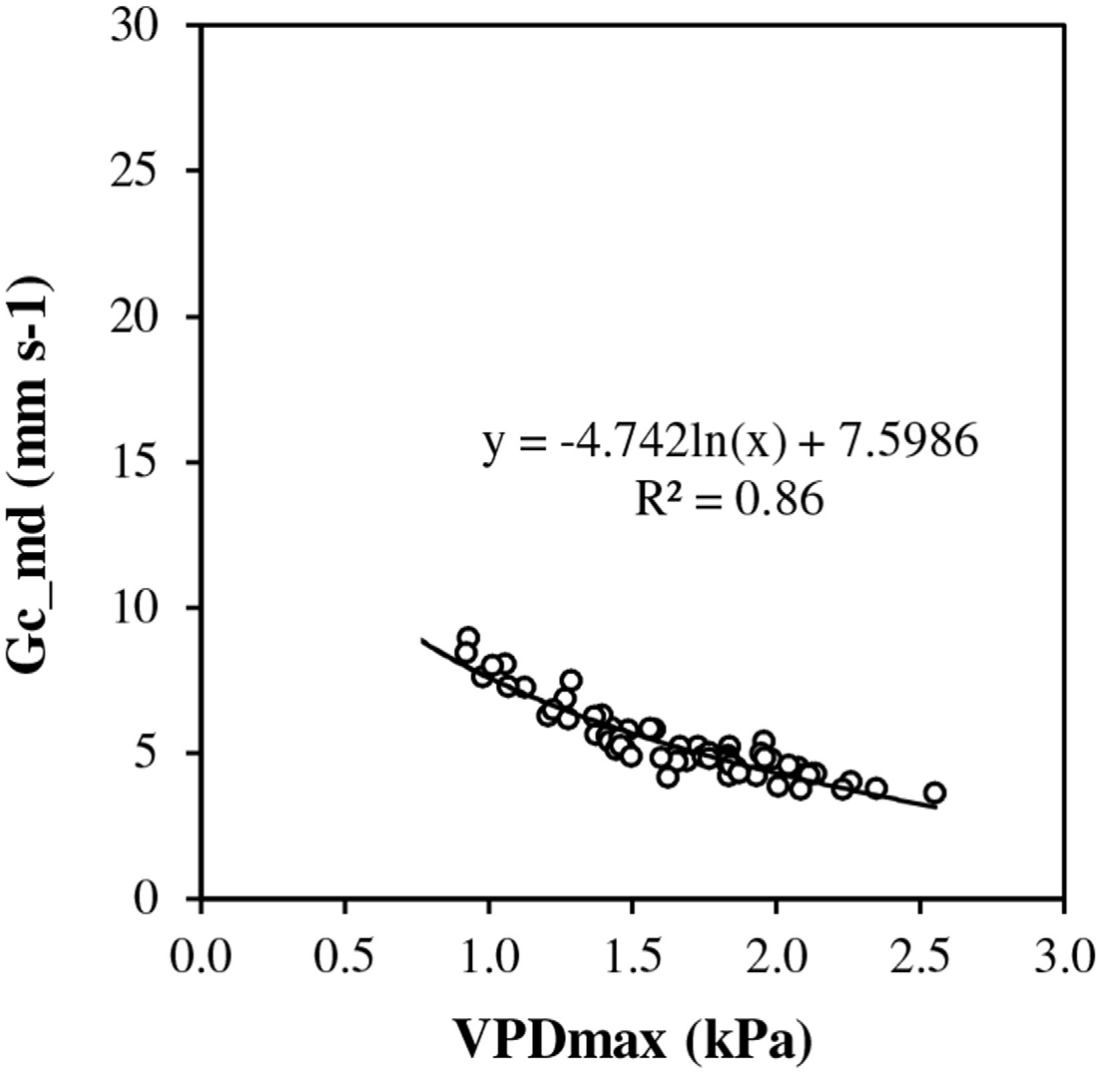
**Midday canopy conductance (*G*_*c*_md_) vs. maximum vapor pressure deficit (*VPD*_max_) at full canopy period and in the absence of soil water stress (*REW* 0–0.6 ≥ 0.5)**.

Under conditions of full canopy and non-limiting soil water (*REW*> 0.5), transpiration plotted vs. *ET0* showed a linear response at low evaporative demand but it exhibited a pseudo-plateau above approximately 2.3 mm d^−1^ (Figure 2A). The average slope of the linear section crossing the origin was estimated as 0.9 (± 0.052). Such a slope corresponds to *rm*, the *T*_max_/*ET0* ratio, in the model framework (Equation [7]). Several values of *T*_max_/*ET0* were above 1.0 at low *ET0*. First, *ET0* is a reference value which does not necessarily represent the maximum *ET* for this particular stand. Second, transpiration could have been overestimated by sap flow measurement, particularly for rainy days and low flow rates. In Figure 2B, *T*_max_/*ET0* was plotted vs. *ET0* discarding y values above 1. The relationship fitted well a Lohammar's function (y = −0.585ln(x) + 1.2492; *R*^2^ = 0.87). The *R*^2^ has little meaning here because the variables were directly related by *ET0* in the calculation. However, Lohammar's function provided better results of *T*_mod_ET0c_ than a linear adjustment. The plot of midday canopy conductance (*G*_*c*_*md*_) vs. *VPD*_max_ confirmed the underlying mechanism of stomatal regulation at increasing *VPD* (Figure 3). According to the fitted Lohammar's function (Lohammar et al., 1980), the reference *G*_*c*_ at 1 KPa and the sensitivity term equal 4.74 mm s^−1^ and 7.6, respectively.

#### LAI

Transpiration estimated by sap flow measurements (*T*_mea_) followed in the expected manner the *LAI* seasonal pattern (Figure 1C) which supports a strong control of transpiration by the *LAI*. However, the soil water availability decreased at the same time in the dry season.

#### REW

**Figure 4:**
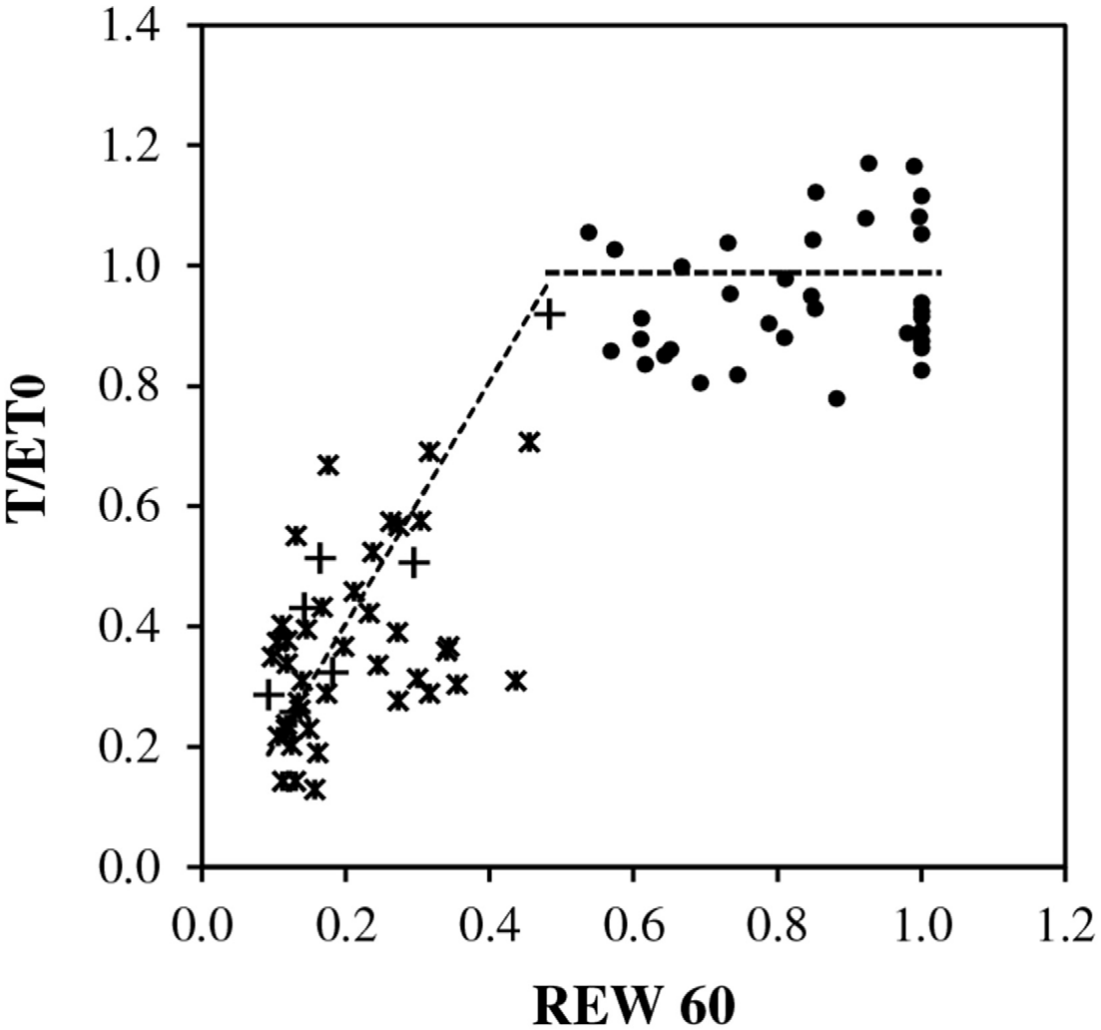
**Relative transpiration (*T/ET0*) vs. relative extractable soil water (*REW*) in top soil (0–0.6 m) during full canopy period (*LAI*_max_), closed circles indicate *REW* ≥ 0.5 and *ET*0 < 2.3 mm d^−1^, asterisks indicate *REW* < 0.5 and *ET*0 ≥ 2.3 mm d^−1^ and plus signs indicate *REW* < 0.5 and *ET*0 < 2.3 mm d^−1^**. Trends are shown as dotted lines, averaged as 1.0 for *REW* ≥ 0 0.5 and as a reduction coefficient for *REW* < 0.5.

The plot of *T/ET0* vs. *REW* showed scatters of points consistent with the assumption of a threshold around *REW* = 0.4 (Figure 4). Above a threshold between 0.4 and 0.5, *T/ET0* exhibited a pseudo-plateau averaging 1.0, with large variability. Below the critical *REW*, a linear decrease toward 0:0 crossed the scatter of points. However, the assessment of the value critical *REW* was approximate due to the lack of soil data between 0.4 and 0.5 *REW*.

### Simulation of transpiration

#### Conditions of simulation

For the calculations described in the Modeling paragraph of Material and Methods:

- *rm* was tested between 0.9 and 1.0 according to Figures 2A, 4, and finally 1.0 was kept.
- *LAI*_max_ was taken equal to 3.9 as measured.
- *REW* was calculated for the top soil (0–0.6 m) with weighting by percentage of fine root length distribution as described in paragraph 2.3.
- The active soil depth and weighting by root distribution were kept constant over the annual cycle.
- *REWc* was taken equal to 0.4 according to BILJOU99 framework and Figure 4.
- *rET0c* was calculated according the function deduced from Figure 2B:
If *ET*0 ≤ *ET*0*c*, *rET*0*c* = 1
If *ET*0 > *ET*0*c*, *rET0c* = *a* ^*^ln(*ET*0) + *b*
with *ET*0*c* = 2.3 mm d^−1^, *a* = −0.585 and *b* = 1.2492.

#### Simulated transpiration

**Figure 5:**
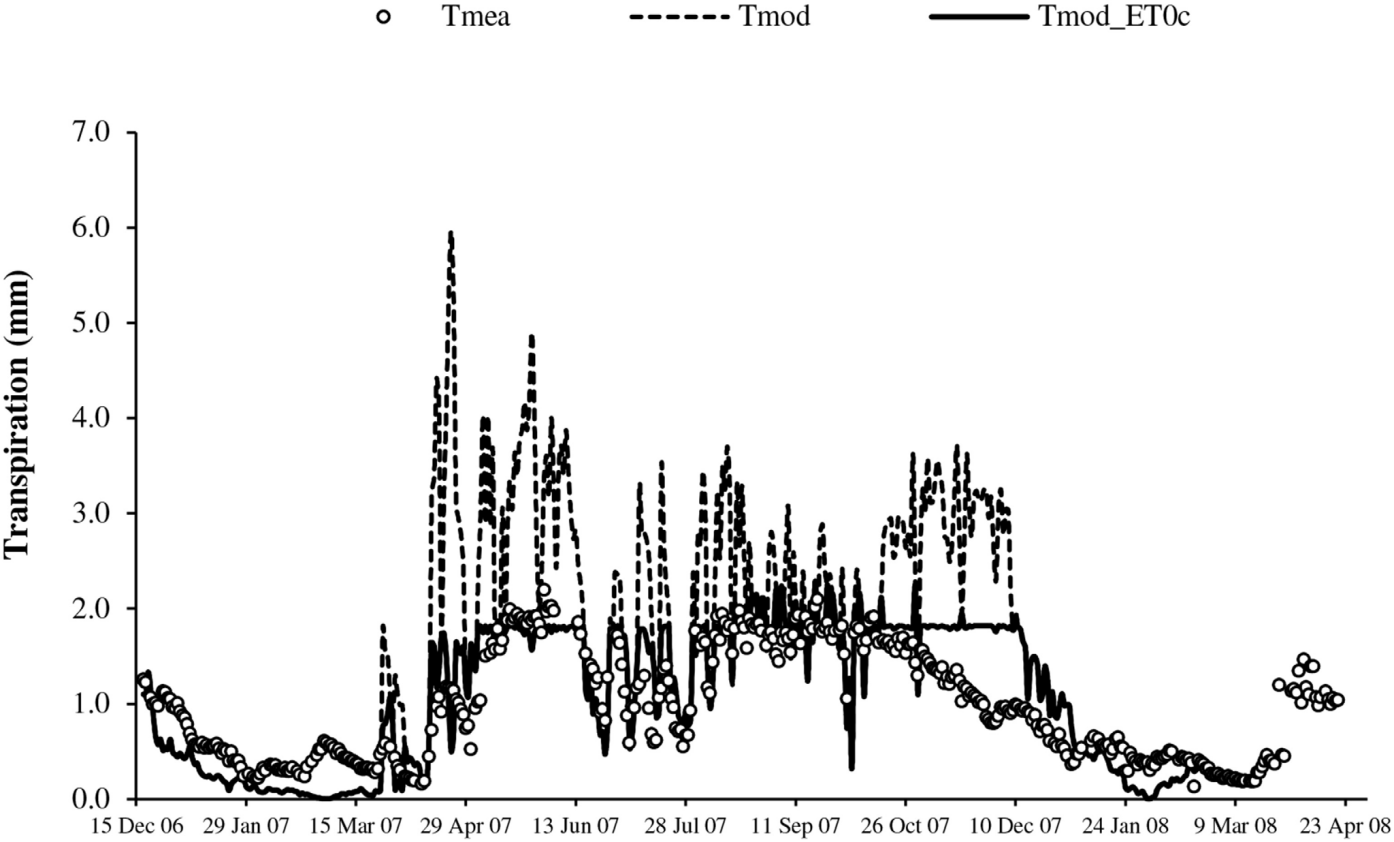
**Seasonal change of transpiration from measurement (*T*_mea_), simulation with original BILJOU99 framework (*T*_mod_) and with evolution including threshold of *ET0* (*T*_mod_ET0c_)**.

The transpirations simulated with the original framework of BILJOU99 (*T*_mod_), largely overestimated during the full canopy period, particularly in April, May, June, August and November, during periods of high evaporative demand (Figure 5). The values simulated with regulation at high *ET0* (*T*_mod_ET0c_) logically better expressed the seasonal change of transpiration; however substantial inaccuracy remained in the dry season with overestimates in November and underestimates in other periods (Figure 5). Table 1 summarizes the previous observations: large errors with *T*_mod_ and the substantial improvement with regulation at high evaporative demand in the rainy season (*RRMSE* < 35%). In the dry season, the errors were substantial (*RRMSE* > 60%); however they are emphasized by the relative expression vs. the low absolute values.

**Table 1:** **Evaluation metrics of simulations of transpiration according to two frameworks: original BILJOU (*T*_mod_), simulation with reduction coefficient of *ET0* (*T*_mod_ET0c_)**.

**Model**	**Observation No**.	***R*^2^**	**Whole year *RMSE(RRMSE)*(mm.d^−**1**^)**	**Wet season *RMSE(RRMSE)*(mm.d^−**1**^)**	**Dry season *RMSE(RRMSE)*(mm.d^−**1**^)**
*T*_mod_	379	0.56	1.06 (1.01)	1.00 (0.67)	1.10 (1.19)
*T*_mod_ET0c_	379	0.77	0.39 (0.56)	0.30 (0.31)	0.45 (0.68)

RMSE; root mean square error and RRMSE; relative root mean square. Wet season from May 1 to October 31, 2007 and Dry season from December 18, 2006 to April 4, 2007 and November 1, 2007 to February 22, 2008.

### Hierarchy between reduction factors of annual transpiration

**Figure 6:**
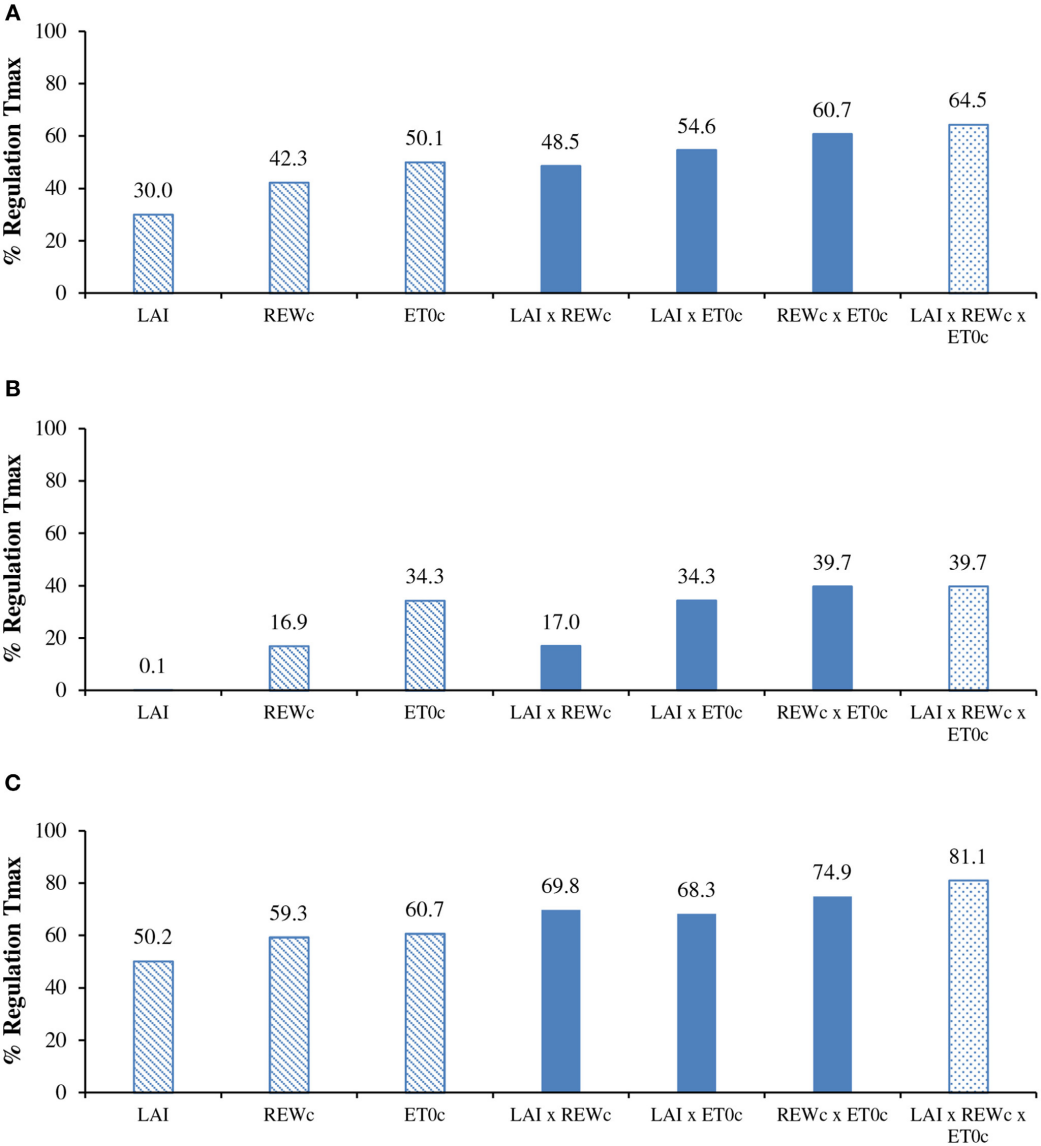
**Regulation of seasonal transpiration (simulated with constraints/maxima) for: (A) the annual cycle; (B) the rainy season; and (C) the dry season, according to reduction factors issued from the *LAI* change, critical relative extractable water (*REWc*) and critical potential evapotranspiration (*ET0c*)**. For details of simulation see paragraphs Modeling and Simulation of transpiration.

On an annual basis, the cumulative measured transpiration (430 mm) was 66% lower than the potential annual transpiration (1247 mm). The regulation was substantial in the rainy season (−39%) and twofold higher in the dry season (−81%). The simulated data provided close estimates of transpiration reduction (−64.5%) when all constraints were considered (*LAI* × *REWc* × *ET*0*c* in Figure 6). When considering only one reduction factor, the *LAI* variation induced the lowest reduction, logically located in the dry season, at the time of defoliation. The impacts of *REWc* and *ET0c* were similar with substantial reduction around −45%, slightly higher for *ET0c* (−50.1%). The *ET0c* constraint was significantly higher than the *REWc* constraint in the rainy season (Figure 6B). It was noteworthy that the overall effect of the *ET0c* constraint alone already represented 76% of the total reduction with the combination of the three factors of constraint. The combination with two factors of constraints supported the proposal that *ET0c* had a larger impact than *REWc*. The small difference in the simulation between *REWc* and *REWc* x *LAI* suggested that the *REWc* constraint included already the *LAI* effect. The *REWc* × *ET0c* interaction induced a reduction equivalent to the reduction with three factors, which confirmed the previous suggestion.

## Discussion

### Basic relationships of BILJOU99 framework

Our results confirmed that the basic relationships of the original framework (Granier et al., 1999) hold except that the regulation of transpiration at high evaporative demand was not well simulated.

#### ET0_c_

Experimental data showed a strong regulation of transpiration under non-limiting soil water at high evaporative demand, when the *ET0* was approximately above 2.3 mm d^−1^. The analysis of the corresponding canopy conductance confirmed a dramatic decrease above *VPD* values equal to 1 kPa. This result supports the previous analysis of Isarangkool Na Ayutthaya et al. (2011) where such a response was related to isohydric behavior with a stable maximal value of the whole tree hydraulic conductance and a stable minimum value of the leaf water potential. Such regulation of transpiration vs. high evaporative demand is known for several species (Pataki and Oren, 2003; David et al., 2004; Ocheltree et al., 2014). However, the sensitivity of this response appears dependent on wood anatomy and hydraulic conductivity. *Hevea brasiliensis* is a diffuse-porous species (Richter and Dallwitz, 2000). And our results follow the general trend that stomatal control vs. high evaporative demand is stricter in diffuse-porous species than in ring-porous species. (Oren and Pataki, 2001; McCulloh and Woodruff, 2012).

#### T_max_/ET0

Below the threshold of critical evaporative demand, the simple and common concept of a stable *rm* ratio between maximal transpiration and reference evapotranspiration under non-limiting conditions held with our data, as *rm* ranged around 1.0 for a maximum *LAI* estimated at 3.89. These values appear relatively high compared to the range of 0.70–0.80 quoted by Granier et al. (1999). However, the latter quotation concerned the ratio vs. *PET* (Potential Evapotranspiration with the Penman equation) which should be higher than *ET0*, the reference evapotranspiration using the Penman-Monteith formula and FAO56 coefficients (Allen et al., 1998). Moreover the accuracy on absolute value of transpiration by sap flow measurement including scaling from tree to stand level is estimated around 20% (Isarangkool Na Ayutthaya et al., 2010).

#### REWc

Our data supported the general assumption of a linear decrease of *T*_max_/*ET0* below a critical value around 0.4 for the *REW* (Granier et al., 1999; Bréda et al., 2006). This threshold of 0.4 was also quoted for other classical expressions of soil water availability, i.e., the plant available soil moisture or PAW (Sadras and Milroy, 1996) or the fraction of transpirable soil water or FTSW (Sinclair et al., 2005).

#### LAI

The seasonal pattern of transpiration followed remarkably the main trend in the *LAI*. However, this observation did not confirm here the well-known control of transpiration by the *LAI* because the *REW* was also decreasing in the dry season. Moreover, simulations in paragraph 3.3 have shown that the decrease in the *REW* was already sufficient to reduce transpiration.

### Simulation of transpiration with model evolution

#### Transpiration

Our results confirmed that including regulation by high evaporative demand in the model largely improved the accuracy in simulation of transpiration. However, substantial errors remained and particularly in the dry season. Besides inaccuracy on estimate of absolute transpiration by sap flow measurement, several points could explain this result with such a simple model. The model relationships were mainly tested in the rainy season and under full canopy conditions and they were applied on the annual cycle. The change in the *LAI* was estimated roughly from litter fall measurements and canopy fullness observations. Temporary water logging could have decreased transpiration in November. Also, stomatal regulation could change with leaf age, particularly during senescence and refoliation (Kositsup et al., 2010). Moreover, the profile of fine root length activity changes in the dry season as shown by Gonkhamdee et al. (2009) while in the simulation, the *REW* calculation used the same soil depth and root profile for weighting. However, at the end, the simulation provided a reasonable indication of trends in seasonal regulation of transpiration which was the objective of the tested framework.

Several models of water balance previously used for rubber tree did not consider atmospheric constraints on transpiration: CROPWAT (Allen et al., 1998), WANULCAS (VanNoordwich and Lusiana, 1999; Guardiola-Claramonte et al., 2010; Boithias et al., 2012). The consequences could be (1) an overestimate of transpiration and root water uptake and (2) a further underestimation of stomatal regulation and consequently an overestimation of C assimilation. In a recent review, Boote et al. (2013) included this process as a current limitation of many crop models. Bregaglio et al. (2014) reported the good performance of a simple approach in arid environments based on transpiration use efficiency that explicitly accounts for the negative impact of vapor pressure deficit on photosynthesis.

A limitation of the reduction factor based on *ET0*c is likely the generality of the relationship and value of *ET0*c which has to be tested in different experimental conditions.

### Diagnosis of the hierarchy between water constraints

The simple framework of simulation has allowed comparing the relative impact of the different constraints on annual and seasonal transpiration. The soil water shortage did not appear as the major water constraint on transpiration in the studied area. High evaporative demand, as expressed by *ET0*, appeared at least of similar importance, if not higher, than soil water shortage. The provided hierarchy is contrary to expectations in this growing area known as climatically under-optimal due to the low amount and variability of rainfall. Clermont-Dauphin et al. (2013) recently provided evidence that soil water shortage was not the main cause of low growth rates in young rubber plantations of North East Thailand and they suggested the importance of high evaporative demand and temporary water logging.

The results from the simulation are directly due to the fact that the sensitivity to air dryness was taken into account in the model. Such a type of response is dependent on the species or variety. We studied the most-planted clone in Thailand (RRIM 600) and the response may change with other varieties of *Hevea brasiliensis*. But the recent of work of Kobayashi et al. (2014) in Cambodia confirmed the sensitivity of canopy stomatal conductance to *VPD* for mature rubber trees of another clone (RRIC 100). However, the details of the response may likely change according to the clone, age and stand characteristics, root development (Devakumar et al., 1999), spatiotemporal acclimation and root proliferation (Liu et al., 2014). We doubt in the generality of the relationship and critical *ET0* used in the framework to simulate sensitivity to air dryness. The results of Sangsing et al. (2004) on young plants of RRIM 600 and RRIT 251 did not show isohydric behavior under water constraints as observed in mature trees. The variability of water relations and sensitivity to air dryness certainly deserves more study.

On the other end, the lower influence of soil water shortage vs. evaporative demand cannot be attributed to a relatively low soil water constraint in the experiment. The growing area of Buri Ram (south of Northeast Thailand) is known as soil water limited with an average annual rainfall of around 1150 mm, which is far below the recommended threshold of 1500 mm. Moreover, the year of study was particularly dry with a rainfall amount of 965 mm. The measurements of soil water availability confirmed the severity of water shortage in both the top soil and deep soil, in the dry season as in the rainy season (Figure 1B).

Defoliation is usually limited to 1–2 months, with the defoliation peak between mid-January and mid-February. However, in dry years such as in the year of study, the period of defoliation could last over 3 months, from January to March. In addition to internal controls, canopy phenology can be influenced by both soil and atmospheric droughts (Eamus and Prior, 2001; Do et al., 2005). These relationships were not taken explicitly into account in the model. It is likely that such inclusions could have changed the results of simulation in the details. However, it is doubtful that they could change completely the conclusion and particularly the fact that impact of atmospheric drought on transpiration regulation was at least of similar importance than soil drought.

## Conclusion

In conclusion, the adapted framework of BILJOU99 had allowed analyzing the relative contribution of soil water shortage and atmospheric drought to the regulation of transpiration on a seasonal scale. This paper provides two main insights. The first stresses the importance of taking into account the direct regulation of transpiration vs. high evaporative demand which often is omitted in simple agro-climatic models. According species, water constraint due to evaporative demand could have been underestimated in previous studies in the sub-humid tropics. The second relies on the interest in simple agro-climatic models to provide a first diagnosis of water constraints on transpiration, in order to help the evolution of cultural choices and practices toward greater sustainability.

## Authors contribution

Jessada Sopharat: data analysis, modeling, paper writing. Supat Isarangkool Na Ayutthaya: experimental design, data collection, data analysis. Frederic Gay: paper writing, editorial advising. Philippe Thaler: paper writing, editorial advising. Sayan Sdoodee: paper writing, editorial advising. Charlchai Tanavud: paper writing, editorial advising. Claude Hammecker: paper writing, editorial advising. Frederic C. Do: experimental design, data collection, supervising the work, modeling, paper writing.

### Conflict of interest statement

The authors declare that the research was conducted in the absence of any commercial or financial relationships that could be construed as a potential conflict of interest.
